# 

**Published:** 1846-09

**Authors:** 


					THE
AMERICAN
JOURNAL
OP
JHental
Science,
PUBLISHED UNDER THE AUSPICES OF THE
American Sortetg of Sental Surgeons.
VOLUME VII.?1846-7.
editors:
CHAPIN A. HARRIS M. D., D. D. S.
AMOS WESTCOTT, M. D .^D'. D. S
EDWIN J. DUNNING.
BALTIMORE:
ARMSTRONG &. BERRY
JOHN W. WOODS, PRINTER.
w
1
A
A\
45"
A1
Virginia Society of Surgeon Dentists.?The fourth annual meeting of the
Virginia Society of Dental Surgeons will be held at Petersburg, Va., on the
second Wednesday in October. Dr. W. W. H. Thackston is to deliver the
opening address. We hope the members of the society generally will be in
attendance on the occasion. The meeting, we have no doubt, will be in-
teresting, as have,indeed, been all the meetings of this association.?Bait. Ed.
Pennsylvania Association of Surgeon Dentists.?This association will hold
its first annual meeting at the Philadelphia Museum, on the first Tuesday
in October, commencing at 7^ o'clock, P. M. As most of the members reside
in the city of Philadelphia, the meeting will, no doubt, be numerously at-
tended.?Bait. Ed.
To Subscribers.?It may be proper that some apology be made to our
subscribers for the delay of this number of the Journal, but we feel confi-
dent that each subscriber will find us a ready excuse w hen informed, that
the entire matter for the Journal had to be made up since the meeting of the
American Society of Dental Surgeons in August last, as well as the trans-
lation of the matter for the library.?Bait. Ed.
mp*.
? W, W. H. Thackston. 6 M 5 00
xfK?3
jfrotessor JJunDar, ^ 3 ? ? ou
B. Gill, ^ - 6? 5 00
?. 8? :*1
5&6 ?
5 & ? " 10 00
1 " IE F. Smyth, 6 m 5 00
1111 81 6 Vol ft 101)0
w w t v/i? n iu uu
? C.W.Harvey. 7 ? 5 00
? S.m, Hambfin, 7 ? 5 .00
? John A. Wright, 6 ?
J? |s B. Gibbs, 6 ?
? A* W. Brown, ager^ ? 66 30
? T.L.Gunn, 6&6 ? 9 80
Spra ;;
iwm
? James Clark. 6 ? 5 00
? W.B. Boss, - 6 ? 5 00
- Jai
^wmi
m f
'mm
ill
LTttaefcston, >45 & '46,
- W. H. Garland, ? '46 & '46, 5 W
Chas. BonsaJL ? '44 to ?46, 10 00
;*
?< N. A. Fisbeii i ? '44 to '46, W WI
? C. O. Cone, ? t; ? '45 to '46, fr.w..
? A. C. Hawcs, ? '43 to ^46, W wa
" N, H. Feun, ? W
?E. Parmly, '46 to ^47, 5 00
Seth P. Miller. ? '44 to '46, 10 00
? w. n. uwineue, ? to ?o, i<# .
! ? Ef J. Canning, ? >45to >47, 7 50
t '48to'4&, ww
? G. W.Juord. lli-
ploma of toe
ima
cietv, and dues, " '45 to 46, 15 00
mmmmdti. ? >45 to '46, 5 00
*45 to '46,
? C. C. AHeru " '44 to '47 12 50
mm B. Hamlin, ? '46 to >47. 2 50
<? VT. I. Hollyman," ?-
" W, Bell&Bgee, ? '44 to '46, W On
I
mmm
.frT -'JA 5
name has been somewhat modified, it may be cons.dered as having en ered npon a newperiod o
it, existence; and its former pubhshers congregate tttemselves.and the professionon,.i
fortune. Its perpetuity is now considered as settled. The prolession will always need a
to its interests, and who is better able to conduct it, than the only regularly organized i
intensive association of dental surgeons in the world. We repeat, tbat we congratulate ourselves
and our professional brethren on an event so aui
a national society and the transfer or ttte journal to that body.
regularly issued, whether its circulation be coextensive with the prolessioa or not. subscripts
be limited, the issues can be made to correspond witHtbe demand, so mat tne expense
to the society may be kept wholly witnm its means.
m The American Journal and Library ot IJentai Science, is now proposed to take an independent
ground, and will hereafter solicit no favours train those members of the profession who are
to the dissemination of periodical information. To others who are fayourably inclined to
nity of knowledge, this journal will not fail to commend itself, as one
fof exposing the impositions of quackery, and imparting to honorable pretensions that kind of
mation which Iheir usefulness and talents should secure.   . r
The journal will be regularly issued on the iirst ot December, Marcfc, June and
annually, at the enhanced price of fiv?
price of subscription may be |>aid to the treasurer ot the society, Dr. C. A. Hams,
, or to either of tbe editors, or any ol the publishers, collaborators or a
the journal. Advance payment is required djt a: special resolution, passed at tne last meeting prpa
society, and is necessary to protect it irorn pecuniary loss, a neglect of wHicU
publishers of the first volume in unexpected embarrassment, it not in ultimate loss.
To save postage, the agents in ail the large cities of the United States, and Great BiSain, wiUM
furnished with copies; to supply subscribers, by such conveyance
where it can be done, even individuals may receive their numbers by private conveyance it UHm
choose.
With these few explanatory remarks,
the profession that no efforts will be spared ta render it every way worthy ot the
EPJ*?
CHAPIN A. HARRIS,
AMOS WlSSTCGTT, 1
EDWIN J.
-Q-ENTS OF THIS JOURNA1.
. ? "T.
?Saxton &Kitts,..........Boston.
John M'Connell, M.?...^Richmond, Va.
. Laird, M. D..  .Columbus, Ga.
B h. 8. Paemw, m .New Orleans.
D * ?????(
ilS?
James Taylor, D.. ... .Cincinnati, Ohio.
A. Vancamp, D. D. S,..... .Nashville, Tcnn.
A. Baldwin, D. D. 8. . ... Alabama.
Nicholas
|pL Jf r ? jg&
&BW?
Benj. Strickland........
B. B. Baoww, M.D
W. R.Scott, D.D.
John A. Cleveland, ..Charleston, s. |
Wm. F. Bason, D. D. S., N. Carolina.1
John Lees, 23 Exchange Arcade, Manchestel

				

